# Four new species of ctenid spiders (Araneae, Ctenidae) from Southeast Asia, with the first description of the female of *Sinoctenuszhui* Marusik, Zhang & Omelko, 2012

**DOI:** 10.3897/BDJ.10.e91350

**Published:** 2022-09-15

**Authors:** Ying Lu, Chang Chu, Zhiyuan Yao, Shuqiang Li

**Affiliations:** 1 College of Life Science, Shenyang Normal University, Shenyang, China College of Life Science, Shenyang Normal University Shenyang China; 2 Institute of Zoology, Chinese Academy of sciences, Beijing, China Institute of Zoology, Chinese Academy of sciences Beijing China

**Keywords:** biodiversity, morphology, new species, taxonomy

## Abstract

**Background:**

The spider family Ctenidae Keyserling, 1877 has a worldwide distribution with 580 species belonging to 49 genera, of which 109 species of six genera are distributed in Southeast Asia.

**New information:**

Four new species of ctenid spiders are described from Southeast Asia: *Anahitamenglun* sp. n. (Yunnan, China), *Bowiehaiphong* sp. n. (Hai Phong, Vietnam), *Bowiemengla* sp. n. (Yunnan, China) and *Bowiezhengi* sp. n. (Yunnan, China). In addition, the female of *Sinoctenuszhui* Marusik, Zhang & Omelko, 2012 (Hainan, China) is described for the first time.

## Introduction

The spider family Ctenidae was established by Keyserling (1876). He placed the family in the suborder Citigradae together with the Lycosidae Sundevall, 1833 and Oxyopidae Thorell, 1869, from which Ctenidae was separated by having two tarsal claws, scopulae beneath the tarsi and three ocular rows arranged in a 2–4–2 pattern (Silva-Dávila 2003). Ctenidae has a worldwide distribution, but the species richness is highest in the tropical forests of South America and Africa (Silva-Dávila 2003, Chu et al. 2022). Jäger (2012) systematically revised the genera *Amauropelma* Raven, Stumkat & Gray, 2001, *Anahita* Karsch, 1879 and *Ctenus* Walckenaer, 1805 from Southeast Asia. Then, in 2022, he established a new lineage, *Bowie* Jäger, 2022, transferred 49 Asian species from *Ctenus* and *Amauropelma* to *Bowie* and published 55 new species of *Bowie* (Jäger 2022). So far, the largest genus of Ctenidae in Southeast Asia has changed from *Ctenus* to *Bowie*.

At present, 580 species belonging to 49 genera of Ctenidae are known worldwide, of which 109 species of six genera are distributed in Southeast Aisa: *Acantheis* Thorell, 1891 (7 spp.), *Amauropelma* (6 spp.), *Anahita* (12 spp.), *Bowie* (82 spp.), *Leptoctenus* L. Koch, 1878 (1 sp.) and *Sinoctenus* Marusik, Zhang & Omelko, 2012 (1 sp.) (Jäger 2022, World Spider Catalog 2022). Amongst these, only 16 species belonging to five genera are distributed in China: *Anahita* (6 spp.), *Amauropelma* (2 spp.), *Bowie* (6 spp.), *Leptoctenus* (1 sp.) and *Sinoctenus* (1 sp.) (Li et al. 2014, Chu et al. 2022, Jäger 2022, World Spider Catalog 2022). Although a large number of new spider species have been reported from China in recent years (Li et al. 2021, Yao et al. 2021, Liu et al. 2022, Lu et al. 2022), the known species of spiders from China are estimated to account for only 5% of the entire Chinese spider fauna (Li et al. 2021, Yao and Li 2021, Hong et al. 2022, Zhu et al. 2022). In this paper, we describe four new species belonging to the family Ctenidae, including three from China and one from Vietnam. Further, we also contribute the first female description and illustrations of *Sinoctenuszhui* Marusik, Zhang & Omelko, 2012 from Hainan, China.

## Materials and methods

Specimens were examined and measured with a Leica M205 C stereomicroscope. Left male pedipalps were photographed. Epigynes were photographed before dissection. Vulvae were treated in a 10% warm solution of potassium hydroxide (KOH) to dissolve soft tissues before illustration. Images were captured with a Canon EOS 750D wide zoom digital camera (24.2 megapixels) mounted on the stereomicroscope mentioned above and assembled using Helicon Focus 3.10.3 image stacking software (Khmelik et al. 2005). All measurements are given in millimetres (mm). Palp and leg measurements are shown as: total length (femur, patella, tibia, metatarsus, tarsus). Leg podomeres were measured on their dorsal side. The distribution map was generated with ArcGIS 10.2 (ESRI Incorporated Company). References to figures in the cited papers are listed in lowercase (fig. or figs.); figures from this paper are noted with a capital letter (Fig.). The specimens studied are preserved in 75% ethanol and deposited in the Institute of Zoology, Chinese Academy of Sciences (IZCAS) in Beijing, China.

Size classes are used according to Jäger (2012), total lengths: small (< 10 mm), medium (10–20 mm), large (20–30 mm), very large (> 30 mm). Palp and leg claw dentition is given according to terminology in Jäger (2012). Arising points of the embolus, median apophysis and conductor in male palps are given as clock-positions of the left palp in a ventral view. Spination pattern is given in two different formulae: in leg patellae and palp, the sum of all spines is listed for the prolateral, dorsal, retrolateral and ventral sides and, when ventral spines are absent, only three digits are listed (Davies 1994, Jäger 2012). In other leg segments, spine positions are given from proximal to distal on each side (prolateral, dorsal, retrolateral, ventral, if present) following Petrunkevitch (1925) and Jäger (2012). Leg formula is given as order of legs according to their length (femur to tarsus measured) in Arabic numbers, for example, 4123. For cheliceral teeth, large and small teeth are separated by “+”, for example, 4 + 1, meaning 4 large and 1 small teeth.

Terminology and taxonomic descriptions follow Jäger (2012) and Jäger (2022). The following abbreviations are used in the descriptions: ALE = anterior lateral eye, AME = anterior median eye, AW = anterior width of prosoma, d = dorsal, OL = opisthosoma length, OW = opisthosoma width, p = prolateral, PL = length of dorsal shield of prosoma, PLE = posterior lateral eye, PME = posterior median eye, PW = width of dorsal shield of prosoma, r = retrolateral, RTA = retrolateral tibial apophysis, TA = tegular apophysis, v = ventral, I–IV = legs I to IV.

The following abbreviations are used in the illustrations: C = conductor, DS = distal retrolateral spine, E = embolus, ET = epigynal teeth, FD = fertilisation duct, RPO = retro-proximal cymbial outgrowth, RTA = retrolateral tibial apophysis, SP = spermathecae, SS = slit sensillum, TA = tegular apophysis.

## Taxon treatments

### Anahita menglun

Yao & Li sp. n.

9DFB952E-C342-5DBD-A2BB-907FE2E6C3DE

6856B6D4-E55F-41D5-9EE7-FDEE238B0D81

#### Materials

- **Type status:** Holotype. **Occurrence:** recordedBy: Guo Zheng; individualCount: 1; sex: male; lifeStage: adult; **Taxon:** order: Araneae; family: Ctenidae; genus: Anahita; **Location:** country: China; stateProvince: Yunnan; municipality: Xishuangbanna; locality: Mengla County; verbatimLocality: Menglun Town, Xishuangbanna Tropical Botanical Garden, *Paramicheliabaillonii* plantation (about 20 yr.); verbatimElevation: 608 ± 11 m a.s.l.; verbatimLatitude: 21°54.200'N; verbatimLongitude: 101°16.923'E; **Event:** samplingProtocol: Collected by hand in leaf litter; year: 2007; month: 4; day: 19–26; **Record Level:** institutionCode: IZCAS-Ar 43472

#### Description

**Male** (IZCAS-Ar 43472): PL 2.1, PW 1.7, AW 0.8, OL 2.2, OW 1.3. Eye diameters and interdistances: AME 0.10, ALE 0.09, PME 0.13, PLE 0.14, AME–AME 0.12, AME–ALE 0.13, PME–PME 0.15, PME–PLE 0.18, AME–PME 0.09, ALE–PLE 0.12, clypeus AME 0.05, clypeus ALE 0.23. Palp and leg measurements: palp 3.0 (0.7, 0.5, 0.6, -, 1.2), I 11.6 (3.0, 1.0, 3.4, 2.9, 1.3), II 9.5 (2.5, 0.9, 2.6, 2.4, 1.1), III 8.6 (2.3, 0.8, 2.2, 2.3, 1.0), IV 13.0 (3.4, 0.9, 3.3, 4.0, 1.4). Leg formula 1423. Spination of palp and legs: palp 130, 110, 1110; femora I p021, d111, r112, II–III p112, d111, r112, IV p112, d111, r111; patellae 000; tibiae I–II v222222, III p11, d11, r11, v222; IV p11, r11, v222; metatarsi I–II v222, III p012, d010, r012, v222, IV p112, r112, v1112. Chelicerae with 3 promarginal, 4 + 1 retromarginal teeth and with elongated narrow patch of about 4 denticles along entire cheliceral furrow. Retromargin of chelicerae close to fang base with one bristle. Leg claws I with 5 secondary teeth and II–IV with 6 secondary teeth. Position of tarsal organ: IV 1.15.

Palp (Fig. 2a–c). Palpal tibia without RTA and intrasegmental sclerite, distally with retrolateral stout spine. Cymbium elongate oval, retrolaterally with slightly stronger bulge than prolaterally. Embolus arising at 5-o’clock-position, long and filiform, running around tegulum, its tip situated distally in 12 to 12.30-o’clock-position. Palp without conductor. Tegular apophysis arising from central tegulum, distally hooked.

Colour (Fig. 9a). Yellowish-brown partly with darker patterns. Dorsal prosoma with two broad lateral bands and marked fovea. Submarginally with narrow band, marginally with thin black line. Sternum and ventral coxae yellowish with darker marks, labium and gnathocoxae yellowish without marks. Chelicerae with two distinct longitudinal bands. Legs yellowish-brown with spots on ventral femora and dark parts mostly on leg IV. Dorsal opisthosoma with distinct light median band. Ventral opisthosoma spotted. Spinnerets yellowish, anterior and posterior spinnerets laterally black.

**Female**

Unknown.

#### Diagnosis

Small Ctenidae (total length male 4.3). The new species can be distinguished from all known congeners by the embolus arising at 5-o’clock-position (Fig. 2b), by the tegular apophysis with hooked tip (Fig. 2a and c) and by the palp having no conductor (Fig. 2a and c).

#### Etymology

The specific name refers to the type locality and is a noun in apposition.

#### Distribution

**Figure 1.**
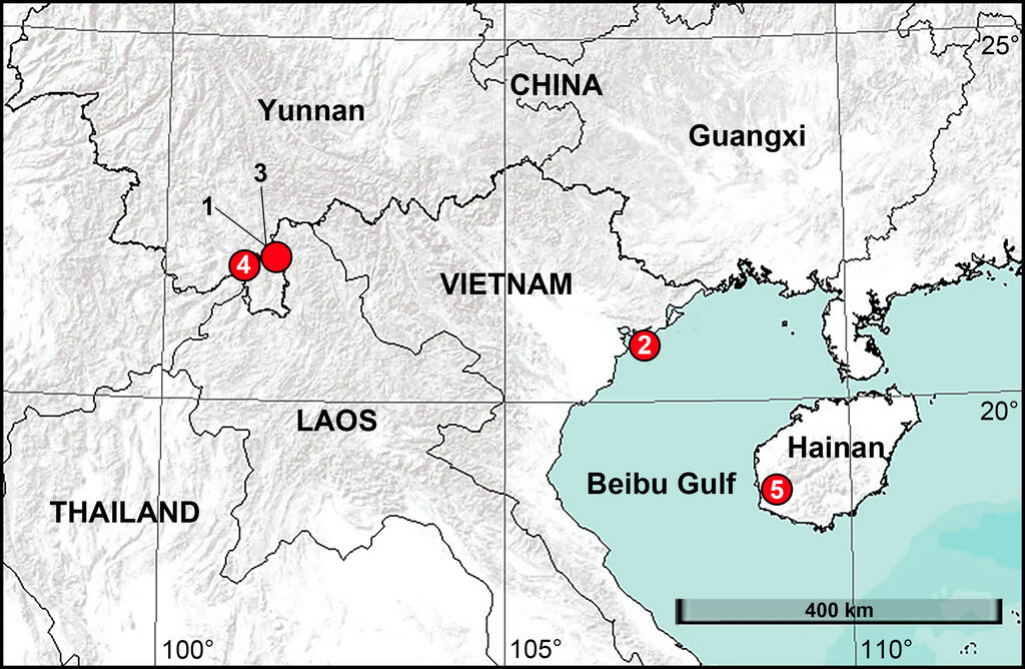
New distribution records of ctenid species from China and Vietnam. 1. *Anahitamenglun* sp. n. 2. *Bowiehaiphong* sp. n. 3. *B.mengla* sp. n. 4. *B.zhengi* sp. n. 5. *Sinoctenuszhui*.

**Figure 2.**
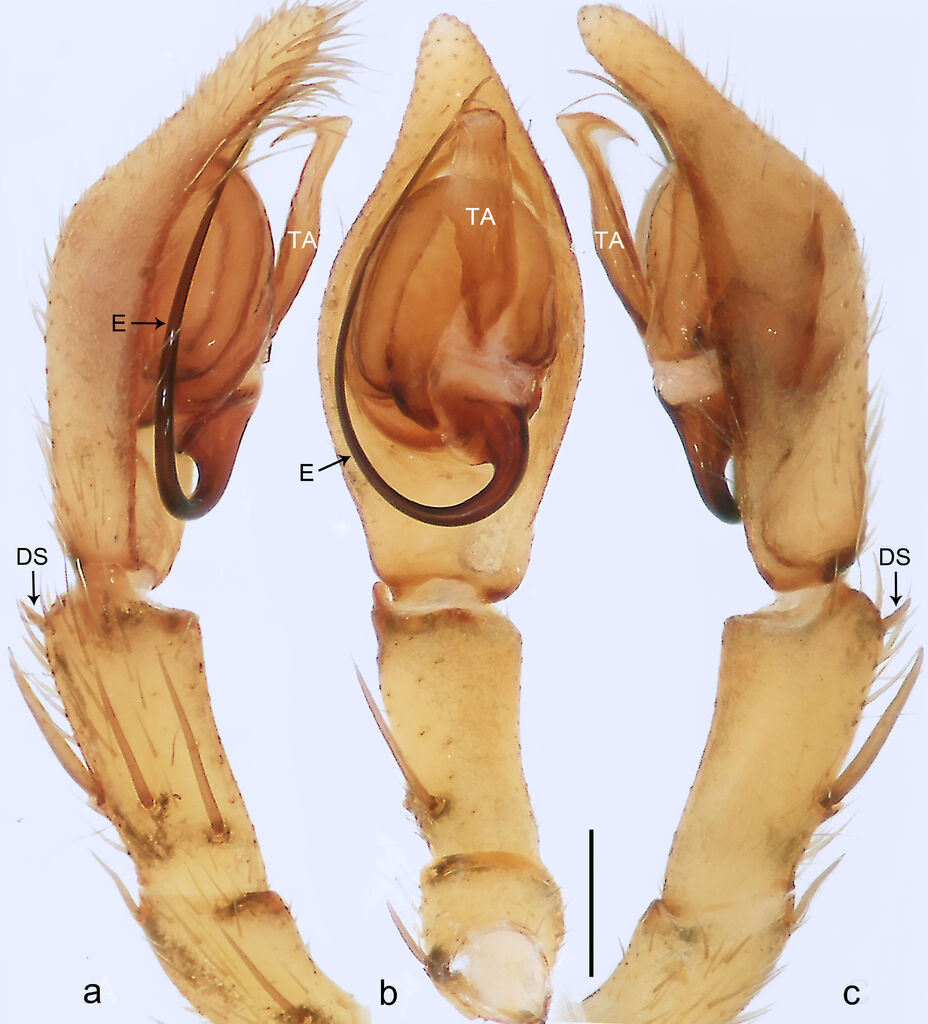
*Anahitamenglun* sp. n., holotype male. **a** Palp, prolateral view; **b** Palp, ventral view; **c** Palp, retrolateral view. DS = distal retrolateral spine, E = embolus, TA = tegular apophysis. Scale bar: 0.20 mm (**a**–**c**).

China (Yunnan, type locality, Fig. 1).

### Bowie haiphong

Yao & Li sp. n.

99299DFF-38CD-5DB4-8193-D16703349858

F569265E-829B-4FDF-B31A-200827E7A5CC

#### Materials

- **Type status:** Holotype. **Occurrence:** recordedBy: Dinh-Sac Pham; individualCount: 1; sex: male; lifeStage: adult; **Taxon:** order: Araneae; family: Ctenidae; genus: Bowie; **Location:** country: Vietnam; municipality: Hai Phong; locality: Vietnam Disturbed Forest, Cat Ba National Park; verbatimLatitude: 20°46.68'N; verbatimLongitude: 106°58.35'E; **Event:** samplingProtocol: Pitfall traps; year: 2007; month: 11; day: 1–30; **Record Level:** institutionCode: IZCAS-Ar 43473
- **Type status:** Paratype. **Occurrence:** recordedBy: Dinh-Sac Pham; individualCount: 3; sex: male; lifeStage: adult; **Taxon:** order: Araneae; family: Ctenidae; genus: Bowie; **Location:** country: Vietnam; municipality: Hai Phong; locality: Vietnam Disturbed Forest, Cat Ba National Park; verbatimLatitude: 20°46.68'N; verbatimLongitude: 106°58.35'E; **Event:** samplingProtocol: Pitfall traps; year: 2007; month: 11; day: 1–30; **Record Level:** institutionCode: IZCAS-Ar 43474–43476
- **Type status:** Paratype. **Occurrence:** recordedBy: Dinh-Sac Pham; individualCount: 1; sex: male; lifeStage: adult; **Taxon:** order: Araneae; family: Ctenidae; genus: Bowie; **Location:** country: Vietnam; municipality: Hai Phong; locality: Vietnam Natural Forest, Cat Ba National Park; verbatimLatitude: 20°46.68'N; verbatimLongitude: 106°58.35'E; **Event:** samplingProtocol: Pitfall traps; year: 2007; month: 4; day: 1–30; **Record Level:** institutionCode: IZCAS-Ar 43477

#### Description

**Male** (IZCAS-Ar 43473): PL 7.9, PW 6.2, AW 3.5, OL 6.2, OW 4.6. Eye diameters and interdistances: AME 0.27, ALE 0.26, PME 0.33, PLE 0.27, AME–AME 0.22, AME–ALE 0.48, PME–PME 0.25, PME–PLE 0.50, AME–PME 0.21, ALE–PLE 0.20, clypeus AME 0.20, clypeus ALE 0.62. Palp and leg measurements: palp 8.4 (3.1, 1.3, 1.3, -, 2.7), I 20.6 (5.8, 3.0, 5.4, 4.8, 1.6), II 19.2 (5.4, 3.4, 4.6, 4.2, 1.6), III 16.1 (5.0, 2.3, 3.3, 4.1, 1.4), IV 22.4 (6.5, 2.4, 5.2, 6.4, 1.9). Leg formula 4123. Spination of palp and legs: palp 161, 100, 1010; femora I p031, d111, r1111, II–III p112, d111, r112, IV p112, d111, r002; patellae 101; tibiae I p110, d111, r1111, v22222, II p110, d111, r110, v22222, III p11, d111, r11, v222, IV p11, d112, r11, v222; metatarsi I–II p111, r111, v222, III p112, d010, r112, v222, IV p112, d010, r112, v2222. Chelicerae with 3 promarginal, 4 retromarginal teeth, without denticles. Retromargin of chelicerae close to fang base with 7 bristles. Ventral tarsi and metatarsi I–II with sparse scopula. Right leg claws I and IV with 3 secondary teeth, claws II and III with 2 secondary teeth.

**Figure 3.**
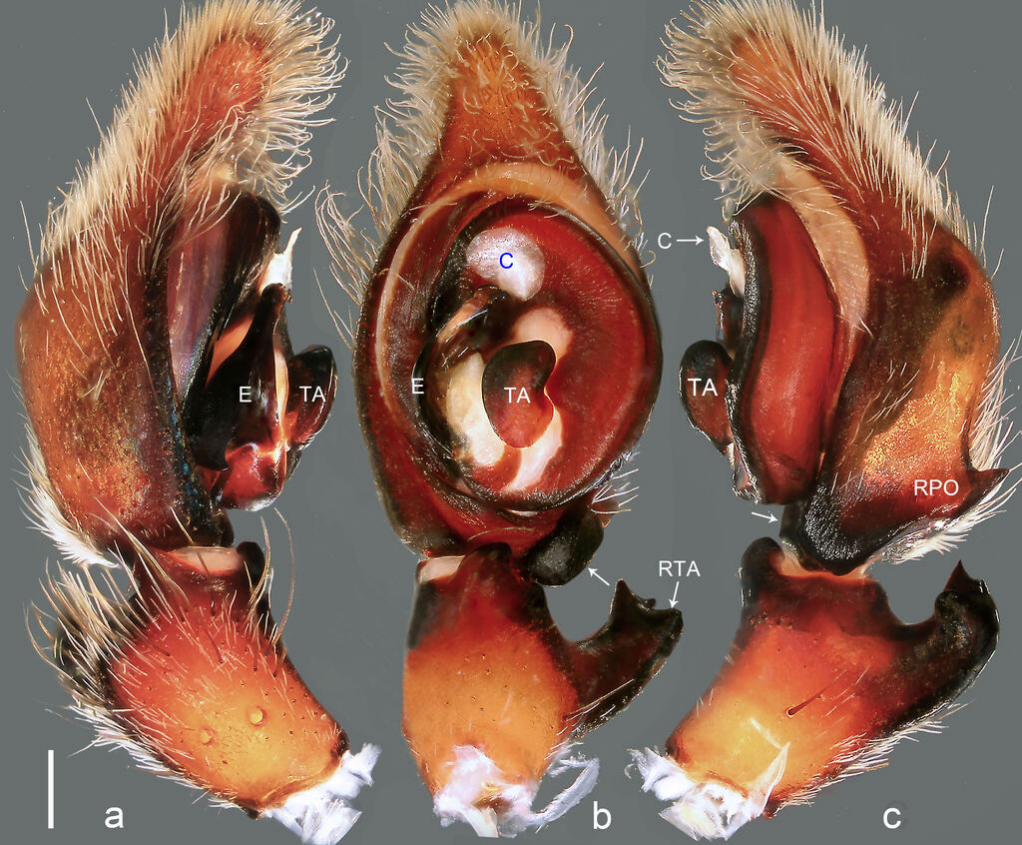
*Bowiehaiphong* sp. n., holotype male. **a** Palp, prolateral view; **b** Palp, ventral view, arrow points at protuberance; **c** Palp, retrolateral view, arrow points at protuberance. C = conductor, E = embolus, RPO = retro-proximal cymbial outgrowth, RTA = retrolateral tibial apophysis, TA = tegular apophysis. Scale bar: 0.20 mm (**a**–**c**).

Palp (Fig. 3a–c). Palpal tibia with strong RTA and with three apices distally. Cymbium tip slightly conical and with pointed retro-proximal cymbial outgrowth. Embolus arising at 7.30-o’clock-position, short, its tip situated in distal half of tegulum (Fig. 8a). Conductor arising at 12-o’clock-position subdistally. Tegular apophysis almost kidney-shaped in ventral view and arising subcentrally from tegulum.

Colour (Fig. 9b). Reddish-brown to yellowish with dark patterns. Dorsal prosoma with characteristic slightly lighter median band, widened behind eyes and with some white hairs, distinctly marked fovea and indistinct radial markings. Sternum and ventral coxae brown, gnathocoxae and labium brown with dark patterns. Chelicerae reddish-brown with longitudinal lines. Leg reddish brown-yellowish. Dorsal opisthosoma yellowish with black patches, most fused into two parallel rows. Lateral opisthosoma yellowish with darker spots. Ventral opisthosoma yellowish with dark patterns; epiandrium and muscle sigilla light. Anterior lateral spinnerets dark, posterior lateral and median spinnerets and anal tubercle light.

**Female**

Unknown.

**Variation**: Paratype males (IZCAS-Ar 43474–43477): PL 7.5–8.0, OL 5.8–5.9.

#### Diagnosis

Medium Ctenidae (total length male 13.3–14.1). The new species is assigned to the *robustus* species-group because of the following characteristics: stout TA; simple stout embolus with broad base and short apical part; presence of retro-proximal cymbial outgrowth; RTA arising medially to subdistally from palpal tibia. Additionally, it resembles *B.candidate* Jäger, 2022 (see Jäger 2022: figs. 254–262, 280–284) by having similar tegular apophysis and embolus, but it can be distinguished by the cymbium with retro-proximal protuberance (arrowed in Fig. 3b and c; absent in *B.candidate*), by the retro-proximal cymbial outgrowth small and pointed (Fig. 3b and c; relatively large and blunt in *B.candidate*) and by the RTA broad in distal part and with three apices distally (Fig. 3b and c; RTA narrow in distal part and with tooth in middle of RTA tip and tibia in *B.candidate*).

#### Etymology

The specific name refers to the type locality and is a noun in apposition.

#### Distribution

Vietnam (Hai Phong, type locality, Fig. 1).

### Bowie mengla

Yao & Li sp. n.

4A61A1FA-61D4-515B-ABF4-2586415AF15F

B9806826-62F1-456B-A624-0B920D4D88B8

#### Materials

- **Type status:** Holotype. **Occurrence:** recordedBy: Guo Zheng; individualCount: 1; sex: male; lifeStage: adult; **Taxon:** order: Araneae; family: Ctenidae; genus: Bowie; **Location:** country: China; stateProvince: Yunnan; municipality: Xishuangbanna; locality: Mengla County; verbatimLocality: Menglun Town, Xishuangbanna Tropical Botanical Garden, Paramicheliabaillonii plantation (about 20 yr.); verbatimElevation: 608 ± 11 m a.s.l.; verbatimLatitude: 21°54.200'N; verbatimLongitude: 101°16.923'E; **Event:** samplingProtocol: Collected by hand in leaf litter; year: 2007; month: 4; day: 19–26; **Record Level:** institutionCode: IZCAS-Ar 43478
- **Type status:** Paratype. **Occurrence:** recordedBy: Guo Zheng; individualCount: 1; sex: male; lifeStage: adult; **Taxon:** order: Araneae; family: Ctenidae; genus: Bowie; **Location:** country: China; stateProvince: Yunnan; municipality: Xishuangbanna; locality: Mengla County; verbatimLocality: Menglun Town, Menglun Nature Reserve, Secondary tropical montane evergreen broad-leaved forest; verbatimElevation: 880 ± 15 m a.s.l.; verbatimLatitude: 21°54.767'N; verbatimLongitude: 101°11.431'E; **Event:** samplingProtocol: Collected by hand in leaf litter; year: 2007; month: 5; day: 4–11; **Record Level:** institutionCode: IZCAS-Ar 43479

#### Description

**Male** (IZCAS-Ar 43478): PL 5.5, PW 4.3, AW 2.2, OL 4.3, OW 2.9. Eye diameters and interdistances: AME 0.22, ALE 0.16, PME 0.28, PLE 0.24, AME–AME 0.14, AME–ALE 0.31, PME–PME 0.24, PME–PLE 0.30, AME–PME 0.13, ALE–PLE 0.20, clypeus AME 0.19, clypeus ALE 0.46. Palp and leg measurements: palp 5.5 (1.9, 0.9, 1.0, -, 1.7), I 19.0 (5.1, 2.3, 4.8, 4.9, 1.9), II 17.0 (4.7, 2.3, 4.2, 4.2, 1.6), III 13.4 (3.8, 1.9, 2.5, 3.7, 1.5), IV 20.2 (5.3, 2.0, 4.5, 6.6, 1.8). Leg formula 4123. Spination of palp and legs: palp 131, 100, 1010; femora I p121, d111, r112, II–III p112, d111, r112, IV p112, d111, r012; patellae 101; tibiae I p100, d111, r110, v22222, II p110, d111, r110, v22222, III–IV p11, d111, r11, v222; metatarsi I–II p111, r111, v222, III p112, r112, v222, IV p112, d010, r112, v2222. Chelicerae with 3 promarginal, 4 retromarginal teeth and with elongated patch of 6 tiny denticles along entire cheliceral furrow. Retromargin of chelicerae close to fang base with 4 bristles. Only tarsi with sparse scopula. Right leg claws I and III with 4, II with 3 and IV with 5 secondary teeth. Position of tarsal organ: I 1.62, II 1.32, III 0.92, IV 1.30.

**Figure 4.**
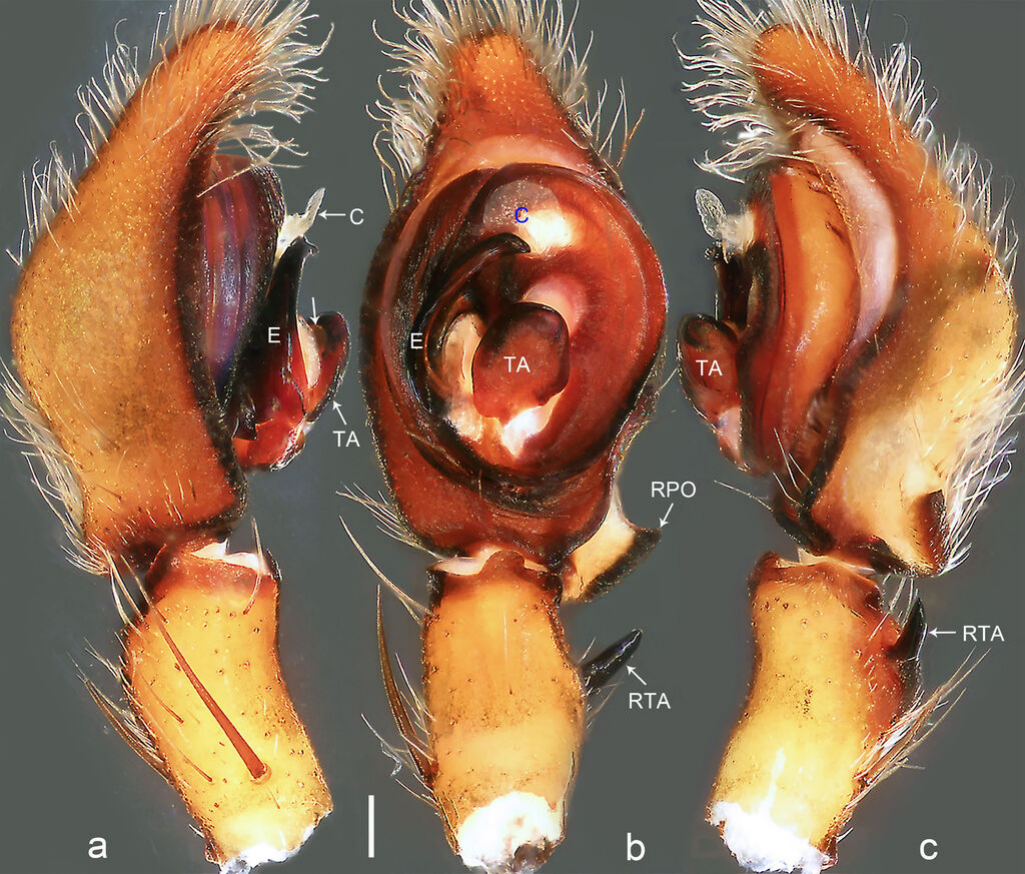
*Bowiemengla* sp. n., holotype male. **a** Palp, prolateral view, arrow points at excavation; **b** Palp, ventral view; **c** Palp, retrolateral view. C = conductor, E = embolus, RPO = retro-proximal cymbial outgrowth, RTA = retrolateral tibial apophysis, TA = tegular apophysis. Scale bar: 0.20 mm (**a**–**c**).

Palp (Fig. 4a–c). Palpal tibia with entirely sclerotised and pointed RTA. Cymbium tip slightly conical, with pointed retro-proximal cymbial outgrowth and the edge of outgrowth sclerotised. Embolus arising at 8-o’clock-position, short, its tip situated in distal half of tegulum. Conductor arising at 12-o’clock-position subdistally. Tegular apophysis arising subcentrally from tegulum, slightly excavated on dorsal side.

Colour (Fig. 9c). Reddish-brown to yellowish with dark patterns. Dorsal prosoma with characteristic slightly lighter median band, widened behind eyes and with some white hairs, distinctly marked fovea and indistinct radial markings. Sternum, ventral coxae and gnathocoxae yellowish, labium yellowish with darker patterns. Chelicerae reddish-brown with longitudinal lines in proximal half and with darker distal half. Leg reddish brown-yellowish. Dorsal opisthosoma yellowish with black patches, most fused into two parallel rows. Lateral opisthosoma yellowish with dark spots. Ventral opisthosoma black with white patches; epiandrium and muscle sigilla light. Anterior lateral spinnerets dark, posterior lateral and median spinnerets and anal tubercle light.

**Female**

Unknown.

**Variation**: Paratype male (IZCAS-Ar 43479): PL 5.2, OL 4.0.

#### Diagnosis

Small Ctenidae (total length male 9.2–9.8). The new species is assigned to the *robustus* species-group because of the following characteristics: stout TA; simple stout embolus with broad base and short apical part; presence of retro-proximal cymbial outgrowth; RTA arising medially to subdistally from palpal tibia; femur III with ventral hump and metatarsus III subproximally with strong cone-shaped ventral hump bearing a spine. Additionally, it resembles *B.fascination* Jäger, 2022 (see Jäger 2022: figs. 230–233 and 263–264) by having similar tegular apophysis and embolus (Fig. 8b), but can be distinguished by the cymbium with pointed retro-proximal outgrowth (Fig. 4b; cymbium with blunt retro-proximal outgrowth in *B.fascination*) and by the RTA thin, pointed and without the tiny tooth at the RTA apex (Fig. 4b and c; RTA relatively broad and with tiny tooth at RTA apex in *B.fascination*).

#### Etymology

The specific name refers to the type locality and is a noun in apposition.

#### Distribution

China (Yunnan, type locality, Fig. 1).

### Bowie zhengi

Yao & Li sp. n.

6C1E72E8-BDD6-50EB-83E3-2E83DEACDCF6

F3EBEC4A-BC46-4CEF-8E6D-237488B6E5DA

#### Materials

- **Type status:** Holotype. **Occurrence:** recordedBy: Guo Zheng; individualCount: 1; sex: male; lifeStage: adult; **Taxon:** order: Araneae; family: Ctenidae; genus: Bowie; **Location:** country: China; stateProvince: Yunnan; municipality: Xishuangbanna; locality: Mengla County; verbatimLocality: Menglun Town, Menglun Nature Reserve, Xishuangbanna Tropical Botanical Garden, Secondary tropical seasonal moist forest; verbatimElevation: 645 ± 17 m a.s.l.; verbatimLatitude: 21°54.718'N; verbatimLongitude: 101°16.940'E; **Event:** samplingProtocol: Pitfall traps; year: 2007; month: 3; day: 1–15; **Record Level:** institutionCode: IZCAS-Ar 43480
- **Type status:** Paratype. **Occurrence:** recordedBy: Guo Zheng; individualCount: 1; sex: male; lifeStage: adult; **Taxon:** order: Araneae; family: Ctenidae; genus: Bowie; **Location:** country: China; stateProvince: Yunnan; municipality: Xishuangbanna; locality: Mengla County; verbatimLocality: Menglun Town, Menglun Nature Reserve, Xishuangbanna Tropical Botanical Garden, Secondary tropical seasonal moist forest; verbatimElevation: 656 ± 15 m a.s.l.; verbatimLatitude: 21°54.984'N; verbatimLongitude: 101°16.982'E; **Event:** samplingProtocol: Pitfall traps; year: 2007; month: 3; day: 16–31; **Record Level:** institutionCode: IZCAS-Ar 43481
- **Type status:** Paratype. **Occurrence:** recordedBy: Guo Zheng; individualCount: 1; sex: female; lifeStage: adult; **Taxon:** order: Araneae; family: Ctenidae; genus: Bowie; **Location:** country: China; stateProvince: Yunnan; municipality: Xishuangbanna; locality: Mengla County; verbatimLocality: Menglun Town, Menglun Nature Reserve, Xishuangbanna Tropical Botanical Garden, Secondary tropical seasonal moist forest; verbatimElevation: 645 ± 17 m a.s.l.; verbatimLatitude: 21°54.718'N; verbatimLongitude: 101°16.940'E; **Event:** samplingProtocol: Pitfall traps; year: 2007; month: 6; day: 19–26; **Record Level:** institutionCode: IZCAS-Ar 43482
- **Type status:** Paratype. **Occurrence:** recordedBy: Guo Zheng; individualCount: 1; sex: female; lifeStage: adult; **Taxon:** order: Araneae; family: Ctenidae; genus: Bowie; **Location:** country: China; stateProvince: Yunnan; municipality: Xishuangbanna; locality: Mengla County; verbatimLocality: Menglun Town, Menglun Nature Reserve, Xishuangbanna Tropical Botanical Garden, Secondary tropical seasonal moist forest; verbatimElevation: 656 ± 15 m a.s.l.; verbatimLatitude: 21°54.984'N; verbatimLongitude: 101°16.982'E; **Event:** samplingProtocol: Pitfall traps; year: 2007; month: 6; day: 1–15; **Record Level:** institutionCode: IZCAS-Ar 43483

#### Description

**Male** (IZCAS-Ar 43480): PL 6.5, PW 5.0, AW 2.3, OL 6.1, OW 4.4. Eye diameters and interdistances: AME 0.23, ALE 0.23, PME 0.31, PLE 0.25, AME–AME 0.19, AME–ALE 0.38, PME–PME 0.21, PME–PLE 0.39, AME–PME 0.19, ALE–PLE 0.20, clypeus AME 0.21, clypeus ALE 0.51. Palp and leg measurements: palp 7.0 (2.5, 1.0, 1.2, -, 2.3), I 17.7 (4.7, 2.3, 4.5, 4.6, 1.6), II 15.8 (4.5, 2.2, 3.7, 3.9, 1.5), III 13.6 (4.2, 2.0, 3.1, 3.1, 1.2), IV 19.6 (5.3, 2.1, 4.6, 5.9, 1.7). Leg formula 4123. Spination of palp and legs: palp 001, 112, 001; femora I p002, d113, r011, II–III p112, d111, r112, IV p112, d111, r002; patellae 101; tibiae I p010, d111, r110, v22222, II p110, d111, r010, v22222, III–IV p11, d111, r11, v222; metatarsi I p111, d001, r111, v222, II p111, d012, r111, v222, III p111, d002, r111, v222, IV p112, d010, r112, v222. Chelicerae with 3 promarginal, 4 retromarginal teeth and with elongated patch of 12 tiny denticles along entire cheliceral furrow. Retromargin of chelicerae close to fang base with 5 bristles. Sparse scopula on all tarsi and metatarsi I–III. Leg claws I, III, IV with 4 and II with 3 secondary teeth. Position of tarsal organ: II 1.32, III 1.02, IV 1.33.

**Figure 5.**
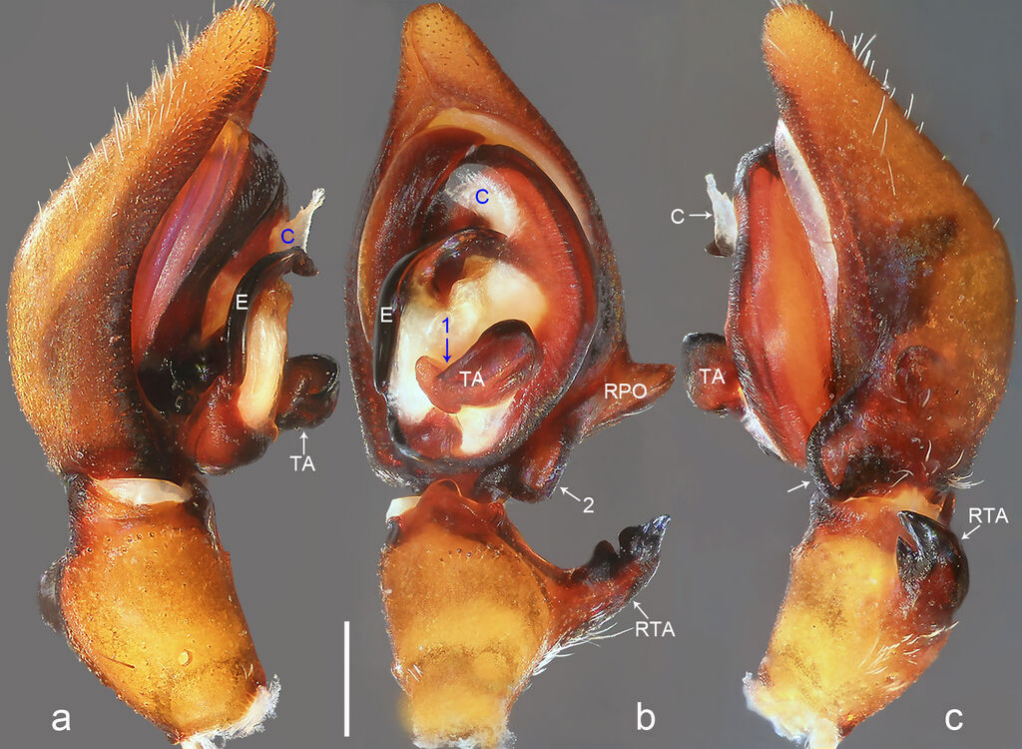
*Bowiezhengi* sp. n., holotype male. **a** Palp, prolateral view; **b** Palp, ventral view, arrow 1 points at concave, arrow 2 points at protuberance; **c** Palp, retrolateral view, arrow points at protuberance. C = conductor, E = embolus, RPO = retro-proximal cymbial outgrowth, RTA = retrolateral tibial apophysis, TA = tegular apophysis. Scale bar: 0.50 mm (**a**–**c**).

Palp (Fig. 5a–c). RTA with sclerotised, triangular subdistal apophysis, distally without distinct teeth and strongly swollen on proximal part. Cymbium tip slightly conical, with retro-proximal outgrowth and retro-proximal protuberance (arrowed 2 in Fig. 5b, arrowed in Fig. 5c). Embolus arising in an 8-o’clock-position from tegulum, short, its tip situated in distal half of tegulum. Conductor arising in a 12-o’clock-position from tegulum. Tegular apophysis arising at 6-o’clock-position from tegulum, distinctly excavated on dorsal side and strongly concave on ventral side (arrowed 1 in Fig. 5b).

**Figure 6.**
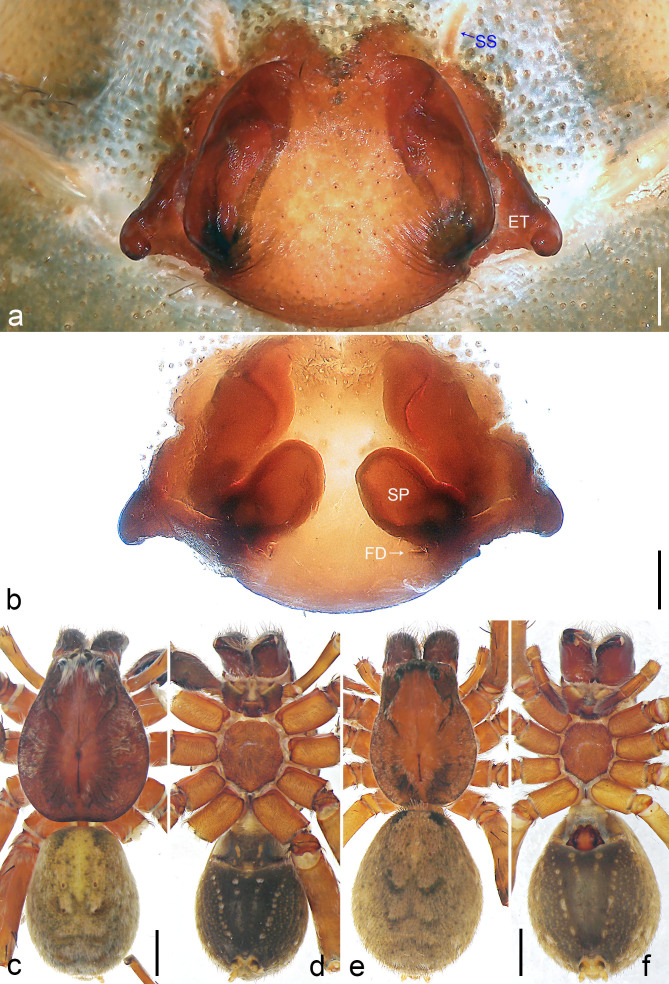
*Bowiezhengi* sp. n. **a** Paratype female, epigyne, ventral view; **b** Paratype female, vulva, dorsal view; **c** Holotype male, habitus, dorsal view; **d** Holotype male, habitus, ventral view; **e** Paratype female, habitus, dorsal view; **f** Paratype female, habitus, ventral view. ET = epigynal teeth, FD = fertilisation duct, SP = spermathecae, SS = slit sensillum. Scale bars: 0.20 mm (**a–b**), 1.00 mm (**c**–**f**).

Colour (Fig. 6c and d). Reddish-brown with darker patterns. Dorsal prosoma with characteristic slightly lighter median band, widened behind eyes, anterior and lateral field with white hairs and with distinctly marked fovea and distinct radial markings. Sternum brown with patterns, ventral coxae yellowish-brown without patterns, labium and gnathocoxae reddish-brown with distal darker spots. Chelicerae reddish-brown. Palps and legs yellowish-brown, without distinct patterns. Dorsal opisthosoma yellowish-brown with black patches, anterior margin and cardiac region with lighter area. Lateral opisthosoma spotted. Ventral opisthosoma dark brown with two posteriorly converging lines of spots and two pairs of distinct spots in the median field close to epigastric furrow. Spinnerets light with anterior lateral spinnerets laterally dark.

**Female** (IZCAS-Ar 43482): PL 6.0, PW 4.6, AW 3.0, OL 6.9, OW 4.7. Eye diameters and interdistances: AME 0.21, ALE 0.22, PME 0.28, PLE 0.27, AME–AME 0.24, AME–ALE 0.41, PME–PME 0.31, PME–PLE 0.49, AME–PME 0.19, ALE–PLE 0.26, clypeus AME 0.16, clypeus ALE 0.50. Palp and leg measurements: palp 5.1 (1.7, 0.9, 1.1, -, 1.4), I 13.2 (3.6, 2.1, 3.4, 2.9, 1.2), II 12.0 (3.5, 1.8, 2.9, 2.8, 1.0), III 11.7 (3.6, 1.9, 2.4, 2.8, 1.0), IV 16.7 (4.4, 2.0, 3.9, 4.9, 1.5). Leg formula 4123. Spination of palp and legs: palp 001, 012, 001; femora I p002, d111, r010, II p112, d111, r111, III p111, d111, r112, IV p001, d111, r102; patellae I–II 000, III–IV 101; tibiae I–II v22222, III–IV p11, d111, r11, v222; metatarsi I–II v222, III p111, d012, r111, v222, IV p112, d010, r112, v222. Chelicerae with 3 promarginal, 4 retromarginal teeth and with elongated patch of 12 tiny denticles along entire cheliceral furrow. Retromargin of chelicerae close to fang base with 6 bristles. Sparse scopula restricted almost entirely to tarsi, only metatarsi I–II with sparse scopula hairs. Palpal claw with 6 secondary teeth, leg claws I–III with 3 and IV with 4 secondary teeth. Position of tarsal organ: I 0.84, II 0.89, III 0.69, IV 0.92.

Copulatory organ (Fig. 6a and b). Epigynal field laterally with two separate long nearly elliptic patches. The anterior part of the median plate is shorter than its posterior part, epigynal teeth nearly triangular, situated submedially at widest part. Internal duct system with two large vulval folds. Spermathecae with two distinctly separated chambers, fertilisation ducts small.

Colour (Fig. 6e and f). As in male, except for: dorsal prosoma with characteristic distinct lighter median band, anterior and lateral field without distinct hairs.

**Variation**: Paratype male (IZCAS-Ar 43481): PL 5.8, OL 5.5. Second paratype female (IZCAS-Ar 43483): PL 6.3, OL 6.9.

#### Diagnosis

Medium Ctenidae (total length male 11.3–12.6, female 12.9–13.2). The new species was assigned to the *bemywife* species-group with the characteristics of retro-proximal protuberance of the cymbium and the transversally orientated TA. Additionally, it resembles *B.yulin* (Yao and Li, 2022) (see Chu et al. 2022: figs. 6–7) by having similar male retro-proximal protuberance of the cymbium, tegular apophysis (Fig. 5a–c) and female epigynal teeth (Fig. 6a), but can be distinguished by the embolus without small membranous process distally (Fig. 5b and Fig. 8c; present in *B.yulin*), by the RTA with sclerotised, triangular subdistal apophysis (Fig. 5b; distally with teeth and strongly swollen on proximal part in *B.yulin*), by the female epigynal median plate narrowed anteriorly (Fig. 6a; the anterior part of median plate is as wide as the posterior part in *B.yulin*) and by the spermathecae not spherical and chamber II small (Fig. 6b; spermathecae both spherical and chamber II large in *B.yulin*).

#### Etymology

The specific name is a patronym in honour of the collector Guo Zheng (Shenyang Normal University, Shenyang, China); noun in genitive case.

#### Distribution

China (Yunnan, type locality, Fig. 1).

### Sinoctenus

Marusik, Zhang & Omelko, 2012

542C303D-7B82-54D4-B8AE-3E91534C7772

#### Description

For the male detail description of *Sinoctenuszhui* Marusik, Zhang & Omelko, 2012, see Marusik et al. (2012) and for the female description, see below.

#### Diagnosis

**Figure 7.**
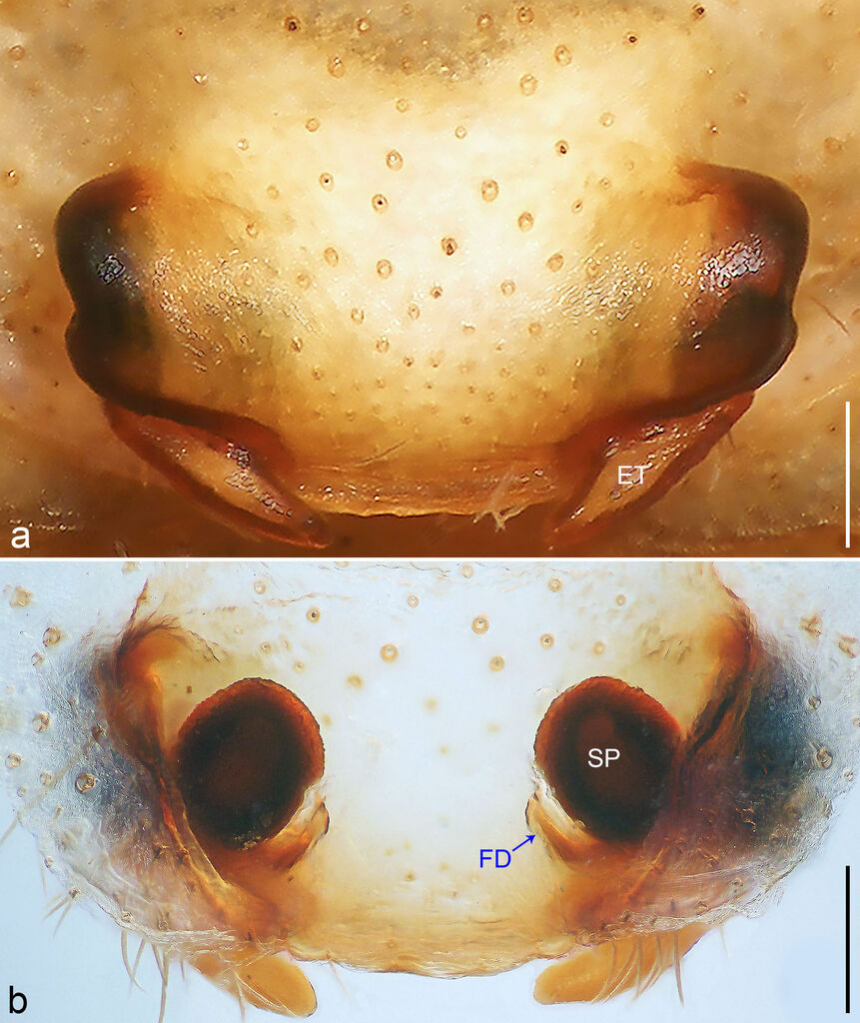
*Sinoctenuszhui* Marusik, Zhang & Omelko, 2012, female. **a** epigyne, ventral view; **b** vulva, dorsal view. ET = epigynal teeth, FD = fertilisation duct, SP = spermathecae. Scale bar: 0.20 mm (**a**–**b**).

It can be distinguished from other genera of the family by the male palp longer than the body (see Marusik et al. 2012: fig. 2), by the male tibia with small spine-like retrolateral apophysis (see Marusik et al. 2012: figs. 11 and 13), by the tegular apophysis covering almost whole bulbus (see Marusik et al. 2012: fig. 9), by the male chelicerae with a ridge on the retromargin part (see Marusik et al. 2012: fig. 6), by the posterior lateral spinnerets with an elongate apical segment (see Marusik et al. 2012: fig. 7), by the anterior part of female epigynal field M-shaped (Fig. 7a), by the epigynal teeth pointed and long, situated posteriorly (Fig. 7a) and by the vulval fertilisation ducts nearly parallel to the margin of spermathecae, pointing medio-anteriorly (Fig. 7b).

#### Distribution

China (Hainan, type locality).

### Sinoctenus zhui

Marusik, Zhang & Omelko, 2012

AFFE3462-BEB2-5884-B418-F9FB3C62270E

#### Materials

- **Type status:** Other material. **Occurrence:** recordedBy: Yuanye Zhou; individualCount: 1; sex: male; lifeStage: adult; **Taxon:** order: Araneae; family: Ctenidae; genus: Sinoctenus; **Location:** country: China; stateProvince: Hainan; locality: Lingshui County; verbatimLocality: Diaoluoshan National Forest Park Resort; verbatimElevation: 920 m a.s.l.; verbatimLatitude: 18°43.505'N; verbatimLongitude: 108°52.104'E; **Event:** year: 2011; month: 4; day: 20; **Record Level:** institutionCode: IZCAS-Ar 43485
- **Type status:** Other material. **Occurrence:** recordedBy: Yuanye Zhou; individualCount: 1; sex: male; lifeStage: adult; **Taxon:** order: Araneae; family: Ctenidae; genus: Sinoctenus; **Location:** country: China; stateProvince: Hainan; locality: Ledong County; verbatimLocality: Jianfengling Town, Jianfengling Ecological Protection Station; verbatimElevation: 820 m a.s.l.; verbatimLatitude: 18°44.415'N; verbatimLongitude: 108°51.802'E; **Event:** year: 2011; month: 5; day: 17; **Record Level:** institutionCode: IZCAS-Ar 43486
- **Type status:** Other material. **Occurrence:** recordedBy: Yuanye Zhou; individualCount: 1; sex: female; lifeStage: adult; **Taxon:** order: Araneae; family: Ctenidae; genus: Sinoctenus; **Location:** country: China; stateProvince: Hainan; locality: Lingshui County; verbatimLocality: Diaoluoshan National Forest Park Resort; verbatimElevation: 920 m a.s.l.; verbatimLatitude: 18°43.505'N; verbatimLongitude: 108°52.104'E; **Event:** year: 2011; month: 4; day: 22; **Record Level:** institutionCode: IZCAS-Ar 43487
- **Type status:** Other material. **Occurrence:** recordedBy: Yuanye Zhou; individualCount: 1; sex: female; lifeStage: adult; **Taxon:** order: Araneae; family: Ctenidae; genus: Sinoctenus; **Location:** country: China; stateProvince: Hainan; locality: Lingshui County; verbatimLocality: Diaoluoshan National Forest Park Resort; verbatimElevation: 920 m a.s.l.; verbatimLatitude: 18°43.505'N; verbatimLongitude: 108°52.104'E; **Event:** year: 2011; month: 4; day: 20; **Record Level:** institutionCode: IZCAS-Ar 43488

#### Description

**Male**: See Marusik et al. (2012).

**Female** (IZCAS-Ar 43487): PL 6.2, PW 4.8, AW 3.0, OL 6.4, OW 4.2. Eye diameters and interdistances: AME 0.20, ALE 0.21, PME 0.29, PLE 0.25, AME–AME 0.24, AME–ALE 0.49, PME–PME 0.36, PME–PLE 0.59, AME–PME 0.18, ALE–PLE 0.28, clypeus AME 0.10, clypeus ALE 0.49. Palp and leg measurements: palp 6.2 (2.1, 1.2, 1.5, -, 1.4), I 14.8 (4.3, 2.3, 3.8, 3.3, 1.1), II 12.5 (3.7, 2.4, 2.2, 3.1, 1.1), III 11.8 (3.6, 1.8, 2.4, 2.9, 1.1), IV 17.2 (4.7, 2.0, 3.9, 5.1, 1.5). Leg formula 4123. Spination of palp and legs: palp 131, 100, 1310, 2120; femora I p021, d111, r111, II p112, d111, r111, III p011, d111, r1111, IV p001, d111, r1111; patellae I–II 000, III–IV 101; tibiae I–II v22222, III p11, d11, r11, v222, IV p11, d111, r11, v222; metatarsi I–II v222, III p111, d012, r111, v222, IV p111, d011, r111, v222. Chelicerae with 3 promarginal, 4 retromarginal teeth, without denticle. Retromargin of chelicerae close to fang base with 5 bristles. Sparse scopula restricted almost entirely to tarsi, only metatarsi I–II with sparse scopula hairs. Palpal claw with 7 secondary teeth, leg claws I, IV with 4, II with 2, III with 3 secondary teeth. Position of tarsal organ: I 0.89, II 0.84, III 0.71, IV 0.91.

Copulatory organ (Fig. 7a and b). Epigynal field laterally with two separate long nearly trapezoidal patches and with two slit sense organs anterior to epigynal plate. The anterior of median plate is longer than posterior, epigynal teeth pointed and long, situated posteriorly. Internal duct system with two small lateral folds. Spermathecae separated by more than their diameter, fertilisation ducts elongately laminar and pointing anteriorly.

Colour (Fig. 9d). Reddish-brown with darker patterns. Dorsal prosoma with characteristic distinct deeper median band, widened behind eyes and with distinctly marked fovea and lateral bands dark reddish-brown. Sternum and ventral coxae dark yellowish-brown without pattern, gnathocoxae and labium deep reddish-brown with distal part lighter, gnathocoxae with slightly light lip. Chelicerae dark reddish-brown. Palps and legs yellowish-brown. Dorsal and lateral opisthosoma yellowish-brown, mottled with black spots. Ventral opisthosoma with triangular yellowish-brown area, median field with darker patches, lateral field brown with black patches. Spinnerets light and anal tubercle light.

**Variation**: Males (IZCAS-Ar 43485, Ar 43486): PL 5.9–6.0, OL 4.6–5.5. Female (IZCAS-Ar 43488): PL 7.7, OL 9.6.

#### Diagnosis

Medium Ctenidae (total length male 10.6–11.4, female 12.6–17.3). The species may be diagnosed by the anterior part of the female epigynal field M-shaped (Fig. 7a), by the epigynal teeth pointed and long, situated posteriorly (Fig. 7a) and by the vulval fertilisation ducts nearly parallel to the margin of spermathecae, pointing medio-anteriorly (Fig. 7b). For the diagnosis of holotype male, see Marusik et al. (2012).

#### Distribution

China (Hainan, type locality, Fig. 1).

## Supplementary Material

XML Treatment for Anahita menglun

XML Treatment for Bowie haiphong

XML Treatment for Bowie mengla

XML Treatment for Bowie zhengi

XML Treatment for Sinoctenus

XML Treatment for Sinoctenus zhui

## Figures and Tables

**Figure 8a.**
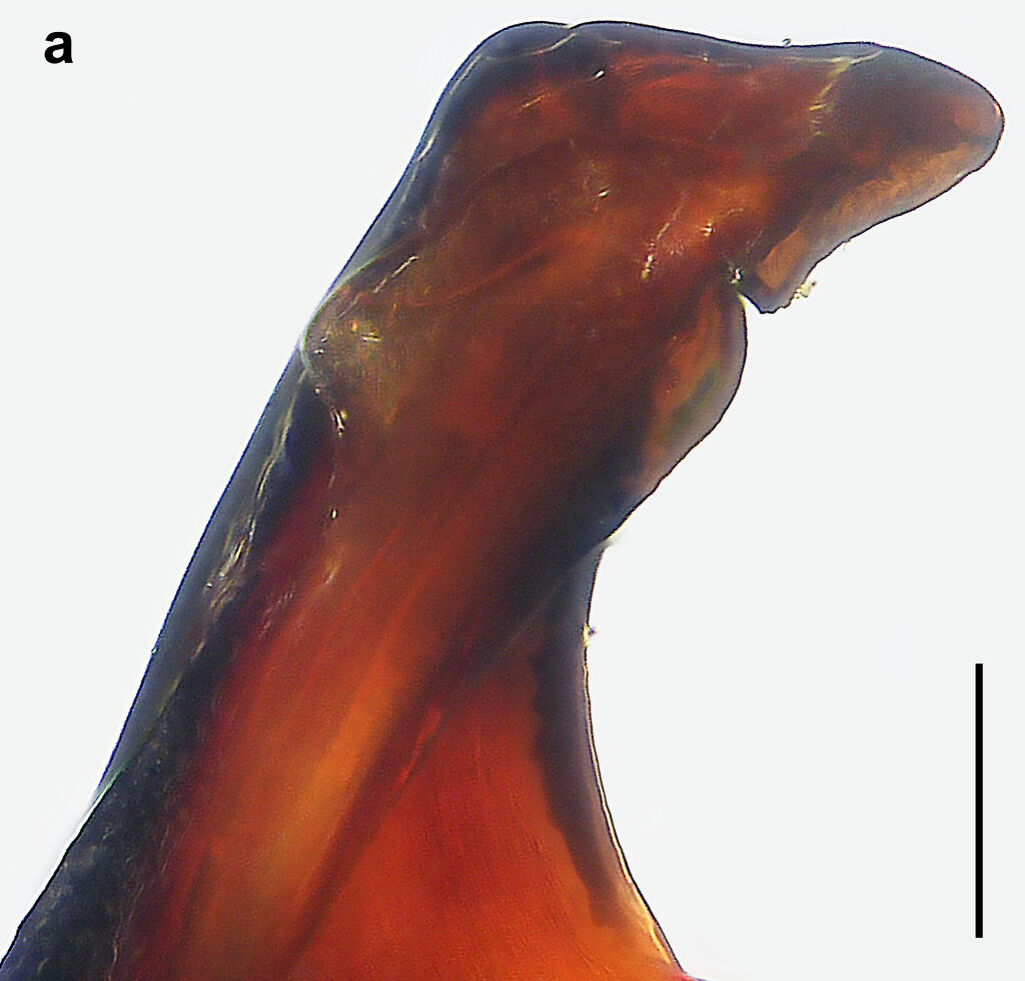
*Bowiehaiphong* sp. n., paratype male. Scale bar: 0.10 mm.

**Figure 8b.**
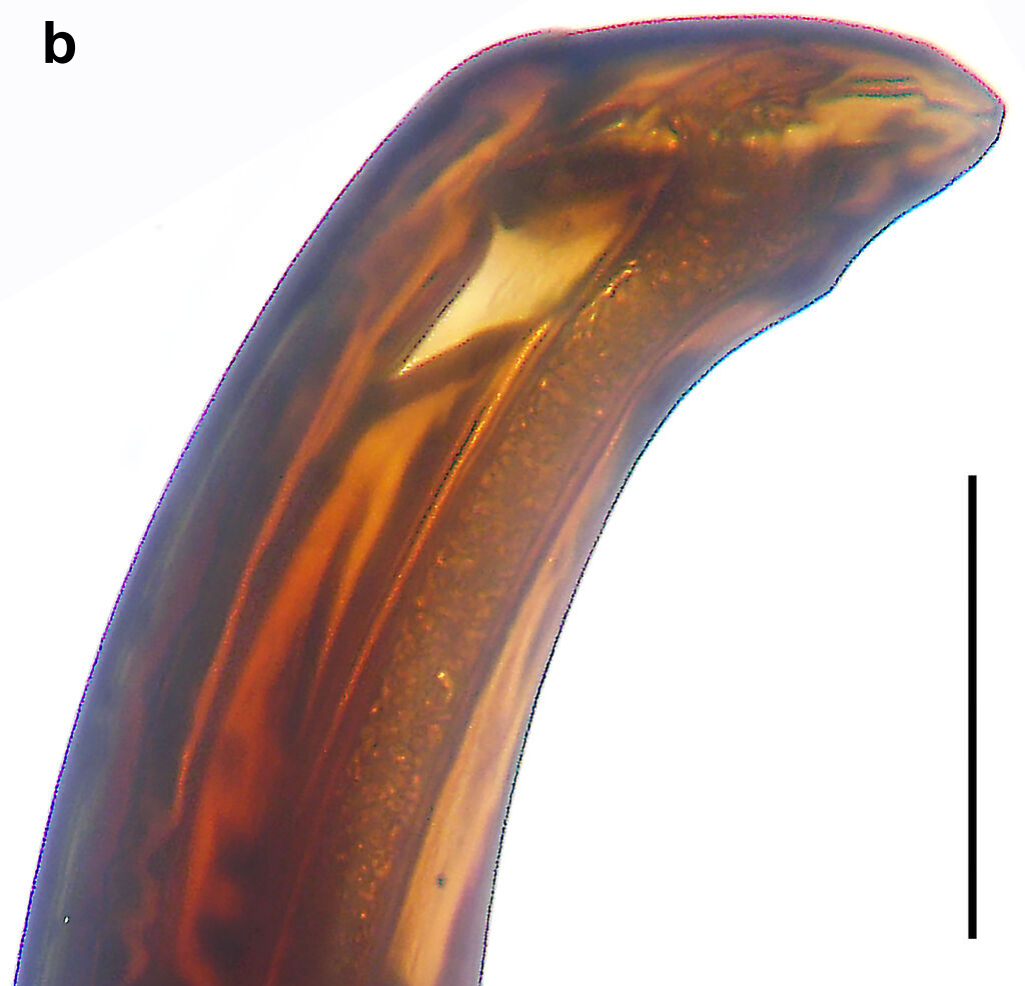
*Bowiemengla* sp. n., paratype male. Scale bar: 0.10 mm.

**Figure 8c.**
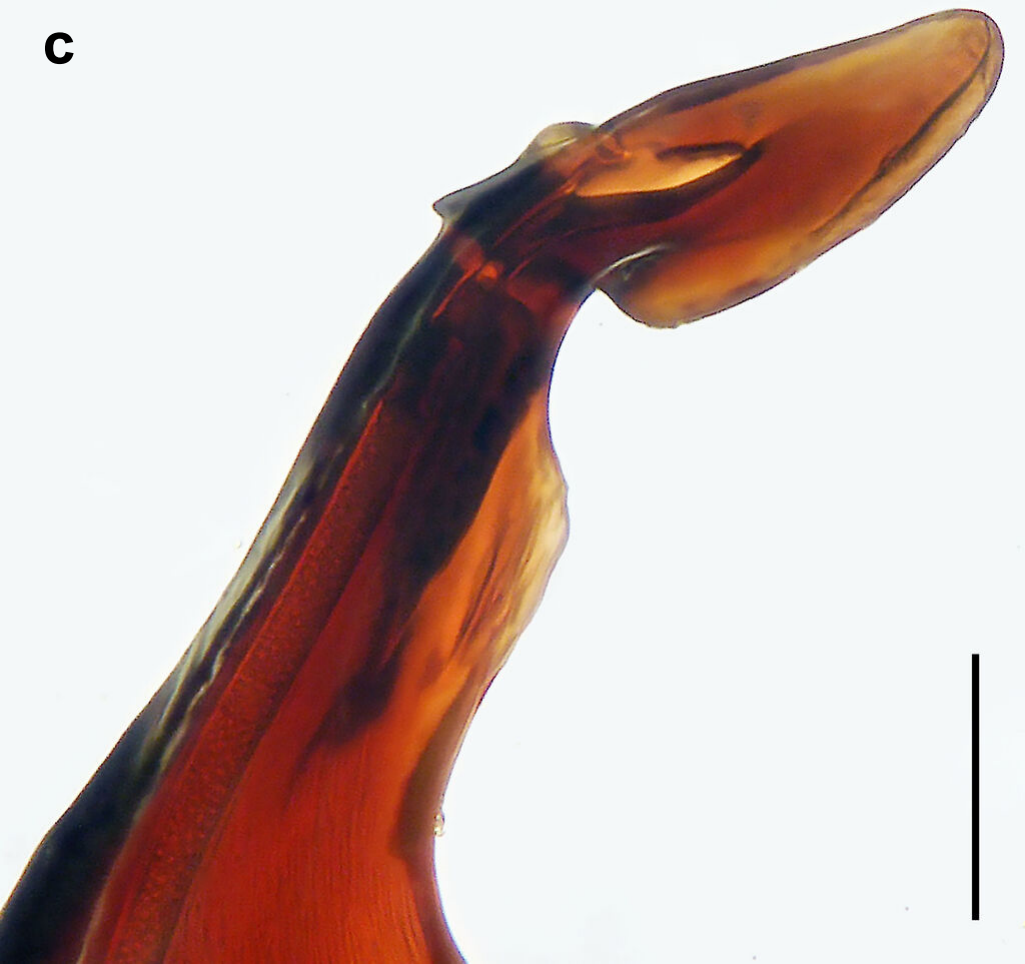
*Bowiezhengi* sp. n., paratype male. Scale bar: 0.10 mm.

**Figure 9a.**
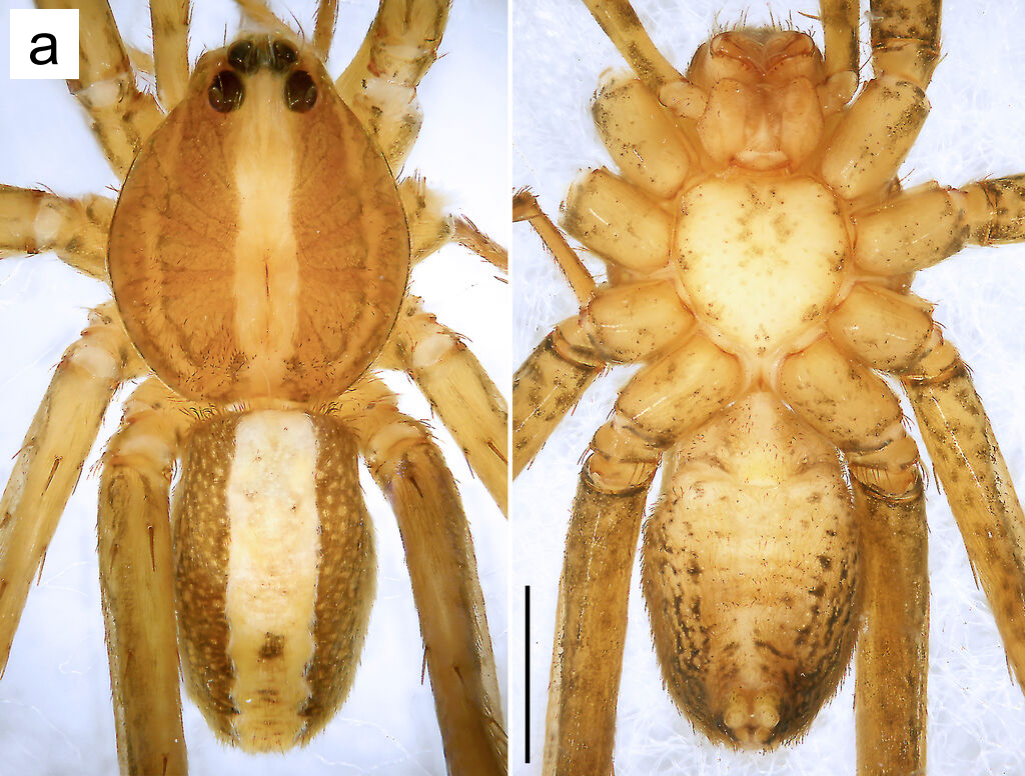
*Anahitamenglun* sp. n., holotype male from China. Scale bar: 1.00 mm.

**Figure 9b.**
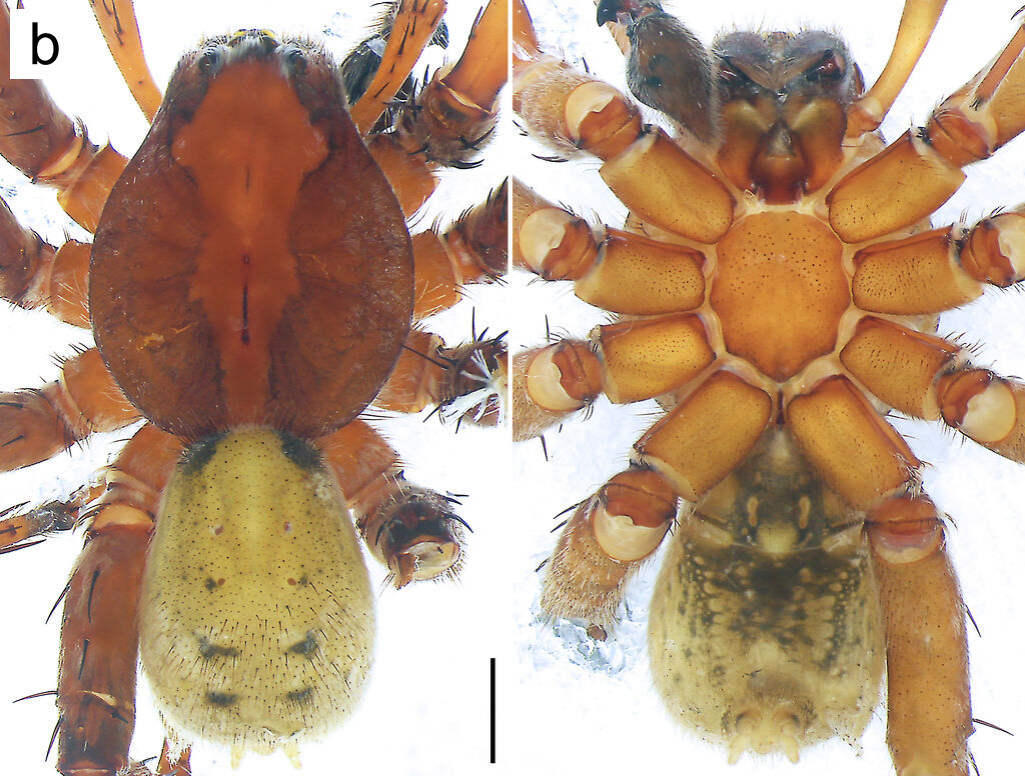
*Bowiehaiphong* sp. n., holotype male from Vietnam. Scale bar: 2.00 mm.

**Figure 9c.**
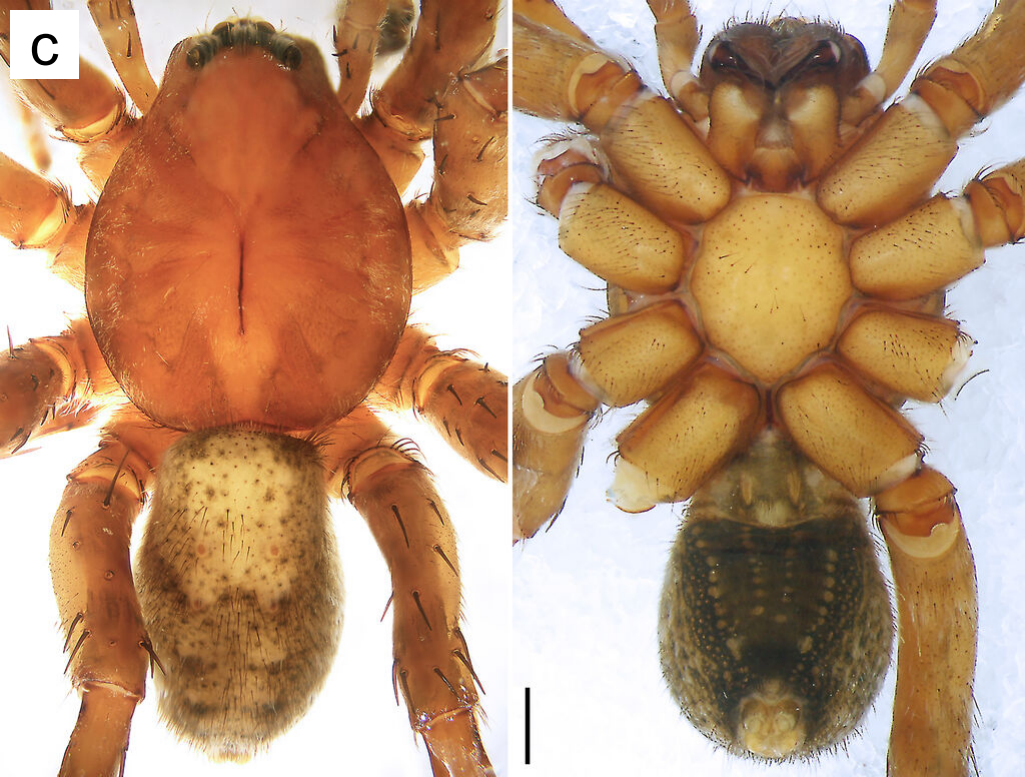
*Bowiemengla* sp. n., holotype male from China. Scale bar: 1.00 mm.

**Figure 9d.**
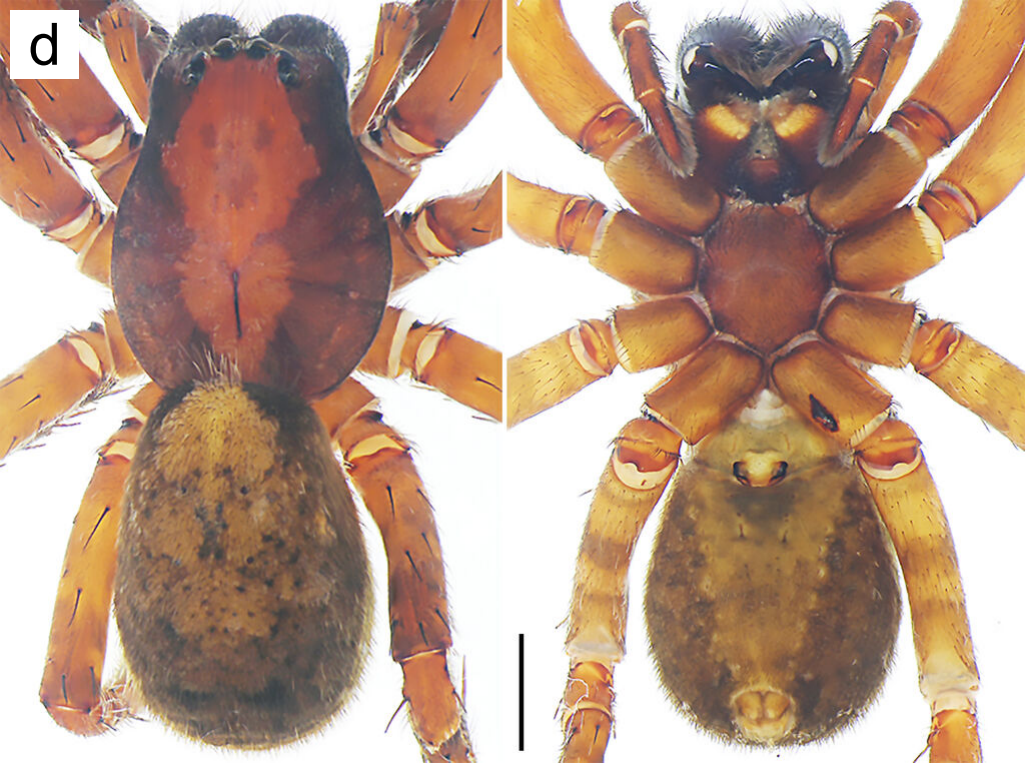
*Sinoctenuszhui* Marusik, Zhang & Omelko, 2012, female from China. Scale bar: 2.00 mm.
